# Reliability and validity of Urdu PARENTS for assessing non-technical skills of paediatric residents in a teaching hospital in Pakistan

**DOI:** 10.1186/s12909-023-04938-2

**Published:** 2023-12-12

**Authors:** Nabila Talat, Rehan Ahmed Khan, Khalid Ahmad Khan, Muhammad Usama Aziz, Warda Tahir, Muhammad Bilal Mirza

**Affiliations:** 1 Department of Paediatric Surgery, University of Child Health Sciences, The Children’s Hospital, Lahore, Pakistan; 2 Department of Medical Education, Riphah International University, Islamabad, Pakistan; 3 Department of Management Sciences, Riphah International University, Lahore, Pakistan

**Keywords:** Assessment, Feedback, Nontechnical skills (NTS), Urdu, PARENTS, Pediatric residents performance

## Abstract

**Purpose:**

The primary objective of our study is twofold. First, we assessed nontechnical skills (NTSs), such as the cognitive, social, and personal skills of postgraduate residents (PGRs), from paediatric caregivers’ perspectives in a paediatric emergency department (PED) of a tertiary care hospital. Second, we evaluated the reliability and validity of the ‘Parents’ Assessment of Residents Enacting Non-Technical Skills’ (PARENTS) instrument in its Urdu-translated version, ensuring its applicability and accuracy in the Pakistani context.

**Materials and methods:**

This mixed-method study used an instrument translation and validation design. We translated an existing instrument, PARENTS, into Urdu, the national language of Pakistan, and administered it to paediatric caregivers in the PED of a tertiary care hospital. We collected data from 471 paediatric caregivers and coded them for analysis in AMOS and SPSS.

**Results:**

The Urdu-translated version of the PARENTS demonstrated reliability and internal validity in our study. The findings from the assessment revealed that paediatric caregivers expressed satisfaction with the knowledge and skill of residents. However, there was comparatively lower satisfaction regarding the residents’ display of patience or empathy towards the children under their care.

**Conclusion:**

The study findings support the validity and reliability of the PARENTS as an effective instrument for assessing the NTS of PGRs from the perspective of paediatric caregivers. With its demonstrated efficacy, medical educators can utilize PARENTS to pinpoint specific areas that require attention regarding the NTS of PGRs, thus facilitating targeted interventions for enhanced patient care outcomes.

## Introduction

To be effective clinicians, medical professionals must possess both technical knowledge and nontechnical skills (NTS). In a comprehensive literature review conducted by YX Tan, AHB Jalal, V Ngai, N Manobharath and TCF Soh [1], eight key NTS were identified: teamwork, communication, asking for help, challenging seniority, task prioritization, decision-making, leadership, and handling stress. Among these, communication plays a pivotal role in establishing trust between doctors and patients.

As part of NTS, communication encompasses the exchange of information, ideas, and emotions between individuals. In healthcare, effective communication is crucial for building rapport, understanding patients’ needs, providing clear instructions, and addressing any concerns or questions. It involves active listening, empathy, clarity, and the ability to convey complex medical information in a way that patients can comprehend. Effective communication is essential for establishing a trusting relationship between patients and physicians [2].

While communication is a broader concept encompassing various aspects of information exchange, counselling represents a specific application of communication skills tailored to provide guidance and support to individuals in need. Patients who have been counselled properly are more likely to follow the treatment plan and hence show better outcomes. A recent study by SS Sajjad, N Sajid, A Fatimi, N Maqbool, N Baig-Ansari and F Amanullah [3] in a TB program in Karachi showed improvement in treatment compliance when doctors properly counselled their patients.

PED is a good place to develop and practice nontechnical skills. PGRs need to develop skills to effectively listen to both the patients and their caregivers, articulate the treatment being administered, address any inquiries they may have, and provide comprehensive postdischarge instructions, which requires developing patience, empathy, and clarity [4]. However, in a public sector healthcare facility setting, where the patient load is high, PGRs can lose patience with paediatric caregivers and vice versa, which can adversely impact outcomes.

Evaluating NTS is especially challenging for supervisors in the PED due to several factors, including high patient loads, time constraints, and administrative commitments. A supervisor can directly observe the soft skills of PGRs, but this can be biased because of the presence of the supervisor [5]. Gaining insight into how PGRs interact with paediatric caregivers or patients in the absence of direct supervision is crucial.

Meaningful feedback on the performance of PGRs can be obtained from several sources, including peers, paramedics, and paediatric caregivers [6]. Pediatric caregivers can serve as valuable assessors for evaluating the NTS of PGRs during the course of treatment [7]. Studies show that residency programmes in paediatric practice (both medical and surgery) can benefit from the feedback of paediatric caregivers as a part of multisource feedback (MSF) [8]. In recent years, medical educationists have pointed to the importance of incorporating the patient’s voice as an important input in enabling the effective delivery of health care [9].

Assessing the NTS of PGRs through lengthy interviews with paediatric caregivers is not practical, primarily due to the significant time investment it would require. Even if qualitative interviews are conducted, converting the qualitative narratives into quantifiable scales for assessing the interactions of PGRs becomes a complex endeavour [10]. However, assessing the NTS of PGRs can be accomplished at discharge by utilizing an objective, easily understood, and comprehensive instrument.

Assessment instruments have been developed by researchers for specific contexts generally related to speciality. In their review of 10 questionnaires for obtaining patient feedback, A Chisholm and J Askham [10] found that only one, SHEFFPAT (UK), was developed for use in paediatric context. However, SHEFFPAT (UK) was developed for general paediatric medicine a context that is very different from the PED environment. PARENTS was developed by KA Moreau, K Eady, K Tang, M Jabbour, JR Frank, M Campbell and SJ Hamstra [11] at the PED of the Canadian Pediatric Academic Health Science Centre. PARENTS has 20 items, of which 18 are closed-ended items and 2 are open-ended items. This instrument has a single factor loading with a Cronbach alpha of 0.95, showing high reliability. This instrument has been previously translated in Thai and used in a general paediatric setting for assessment of NTS of PGRs by paediatric caregivers [12].

The primary objective of this study encompasses two key aspects: first, the translation of the PARENTS into Urdu, and second, its validation in a PED setting in Pakistan. This validation process aims to establish the reliability and applicability of the Urdu version of PARENTS for assessing NTS of PGR by paediatric caregivers. Through this study, we seek to affirm the efficacy of PARENTS as a robust tool for measuring these skills in a culturally diverse healthcare environment.

## Materials and methods

This study aimed at instrument translation and validation. We collected and analysed both qualitative and quantitative data using a mixed method design as defined by KE Schifferdecker and VA Reed [13] with regard to application in medical education. We reviewed the literature to identify an instrument that can be used for assessing the NTS of residents and selected the PARENTS, which was developed in Canada in the English language. We translated PARENTS from English to Urdu following the process specified by M Birbili [14]. A bilingual expert initially translated the instrument, followed by a back-translation to check for inconsistencies. An expert panel then reviewed both versions, making crucial adjustments. We pilot tested the translated instrument after obtaining ethical approval from the hospitals’ Institutional Review Board (IRB approval number ERC/10/20/11).

**Fig. 1:**
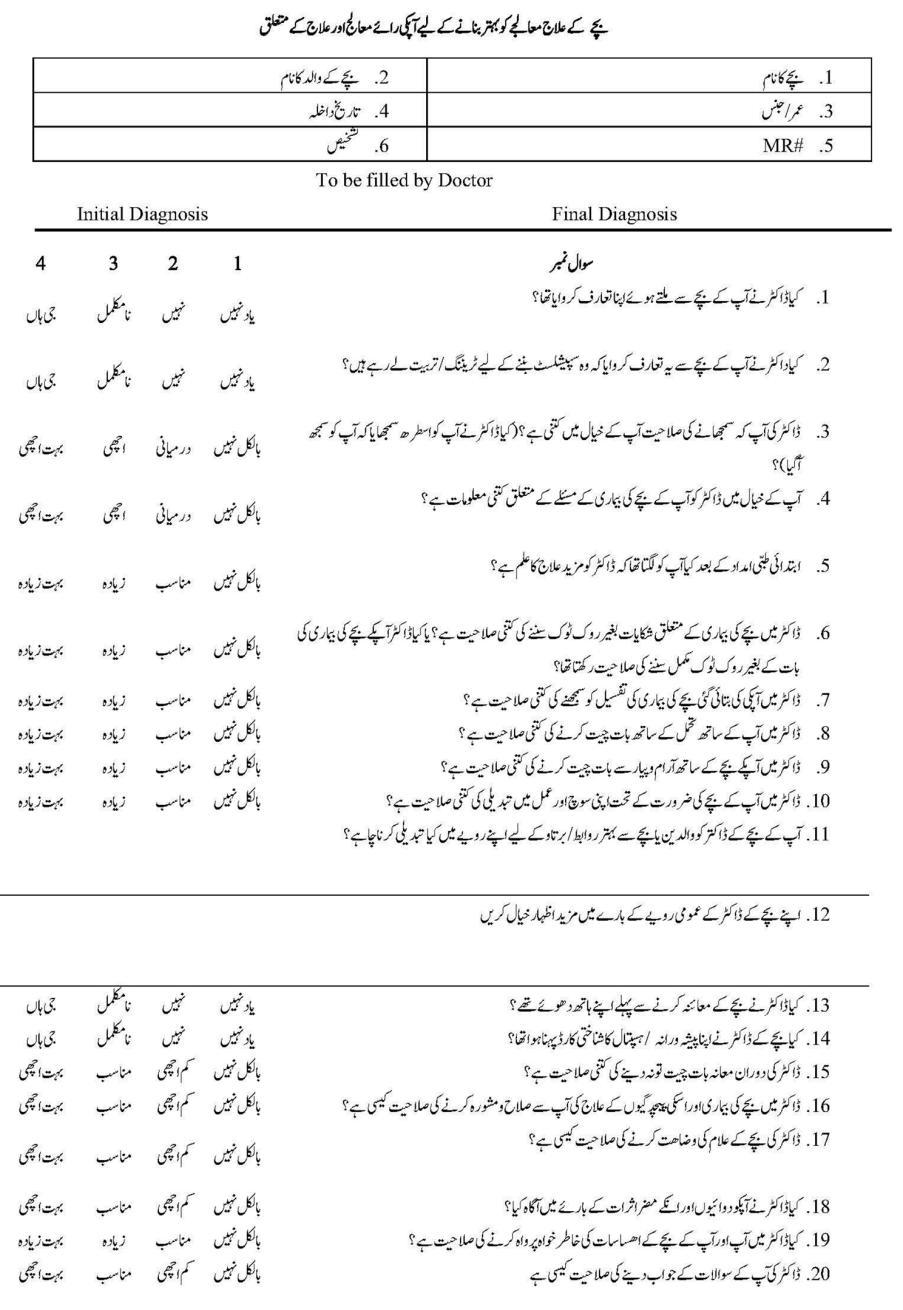
Urdu version of PARENTS

The cultural adaptation of the PARENTS instrument extends beyond linguistic translation, from English to Urdu (see Fig. 1), to encompass the nuances of cultural expression and understanding in healthcare settings. Such cultural sensitivity in instrument validation is crucial for maintaining the tool’s relevance and effectiveness, as supported by research emphasizing the importance of cultural appropriateness in instrument translation and validation in healthcare contexts [15].

Our study included a pilot phase with 30 paediatric caregivers to test the questionnaire’s comprehensibility and response format. Our primary objective for the pilot study was to assess the clarity and ease of use of the questionnaire and to ensure its suitability for the target population with diverse literacy levels. During pilot testing, we noticed participant confusion with the original 5-point Likert scale, which led us to revise it to a 4-point format. This was based on R Garland [16] recommendation on minimizing social desirability bias, where respondents may not truly express their opinions but rather opt for socially desirable responses. Additionally, due to the varied literacy levels, we engaged two residents as enumerators to clearly explain the questionnaire items and accurately record responses. The questionnaire typically necessitated a 15–20 min completion timeframe, inclusive of allaying caregivers’ apprehensions regarding any potential negative implications of their responses on the treatment of their patients.

In this study, we adhered to a methodological guideline suggesting a participant-to-item ratio of 20:1 for confirmatory factor analysis (CFA), a practice considered robust for scale validation studies. Hence, given our 20-item questionnaire, we aimed for a minimum sample size of 400 [17]. This approach was intended to bolster the reliability and validity of our research findings.

We conducted the study at the Paediatric Emergency Department of the Children’s Hospital and University of Child Health Sciences Lahore in both medical and surgical sections in March 2021 with a two-week time window for data collection to ensure minimum variability in the data collection environment. We interviewed the caregivers of the patients admitted to the PED who were discharged after spending 24 h in the facility. They were interviewed after obtaining verbal consent (voluntary basis) by the enumerators, who accordingly filled out the questionnaire.

We were able to obtain 471 completed responses in the sample period, which exceeded our minimum target for the study. In all, we collected assessments on 80 PGRs. In our study, demographic data collection encompassed departmental affiliation (medicine or surgery), patient gender, age, and disease nature. We used this approach to ensure a comprehensive representation across various demographic categories. The inclusion of these diverse demographics was important in enhancing our ability to generalize the findings, as it provided a deeper understanding of the population’s composition and dynamics. This breadth in data collection is essential for reinforcing the robustness and wider applicability of our research outcomes.

We entered the collected data and analysed it in the computer software SPSS version 22.0 (Statistical Package for Social Sciences) to check reliability using Cronbach’s alpha test and in AMOS v.24 to check validity using confirmatory factor analysis.

## Results

We carried out descriptive statistics on all items except open-ended items (Items 11 and 12) to determine the mean, standard deviation, kurtosis, and skewness.

The sample was composed of patients with a distribution across distinct age groups: infants (age 1 month-1 year) represented the largest group (n = 161, 34.2%), followed by neonates (age up to 1 month) (n = 121, 25.7%), school-going children (age above 3 years) (n = 111, 23.6%), and preschoolers (age 1 to 3 years) (n = 78, 16.6%). The gender distribution of the sample comprised males (n = 280, 59.4%) and females (n = 191, 40.6%). In terms of disease severity, 39.5% of the patients were categorized as severe (n = 186), 32.9% as moderate (n = 155), and 27.6% as mild (n = 130) (see Table 1).

**Table 1:** Disease Severity Classification of Patients

Disease Severity	Disease	Number of Patients (n)	Percentage (%)
Severe	Severe Celiac Disease Crisis, Cloacal Exstrophy, Enteric Perforation, Febrile Seizures, Gastroschisis, Head Injury, Infective Endocarditis, Malignancy, Meningitis, Necrotizing Fasciitis, Omphalocele, Pleural Effusion, Pneumonia, Sepsis, Tension Pneumothorax, Testicular Torsion, Wilms’ Tumor	186	39.5%
Moderate	Asthma, Bone Fracture, Bronchiolitis, Burn, Cholestasis, Corrosive Intake, Cow’s Milk Protein Allergy, Epididymoorchitis, Esophageal Web, Exploratory Laparotomy + Adhesiolysis, Hemolytic Anemia, Hepatitis, Intussusception, Liver Cyst, Meconium Ileus, Metabolic Seizures, Perforated Appendix, Septic Ileus, Stoma Prolapse, Stomal Diarrhea, Tuberculosis Abdomen	155	32.9%
Mild	Abscess, Appendicitis, Band Obstruction, Foreign Body in Nose, Inguinal Hernia, Intestinal Atresia, Laceration, Malrotation, Prolapsed Rectal Polyp, Psoas Abscess, Umbilical Polyp	130	27.6%

Patients from the Surgery department constituted a slight majority (n = 249, 52.9%) compared to those from the Medicine department (n = 222, 47.1%). The demographics demonstrated a broad representation of patient characteristics within the study sample (see Table 2).

**Table 2:** Demographic Characteristics of the Patient Sample

Characteristic	Category	Frequency (n)	Percentage (%)
Age Group	Neonate	121	25.7%
	Infant	161	34.2%
	Pre-schooler	78	16.6%
	School-going	111	23.6%
Gender	Male	280	59.4%
	Female	191	40.6%
Disease	Severe	186	39.5%
	Moderate	155	32.9%
	Mild	130	27.6%
Department	Medicine	222	47.1%
	Surgery	249	52.9%

Cronbach’s alpha analysis for 18 closed-ended items was performed in SPSS, and we obtained a value of 0.884, which is considered highly reliable [18]. We checked the internal validity of the PARENTS scale via a Pearson correlation analysis of the 18 items (see Table 3).

**Table 3:** Pearson Correlation of Items Within PARENTS

Item	Pearson Correlation
1. Did the resident introduce him/herself when meeting you and your child for the first time?	0.293**
2. Did the resident identify him/herself as a resident?	0.167**
3. How would you assess the resident’s skill to explain things in a way that you could understand?	0.641**
4. How would you assess the resident’s skill to enter the room with some basic knowledge of your child’s condition?	0.727**
5. How would you assess the resident’s skill to determine next steps about care or treatment with you, including any follow-up plans?	0.639**
6. How would you assess the resident’s skill to listen to you and speak without interruption?	0.632**
7. How would you assess the resident’s skill to understand what you had to say?	0.689**
8. How would you assess the resident’s skill to interact with you comfortably?	0.780**
9. How would you assess the resident’s skill to interact with your child comfortably?	0.734**
10. How would you assess the resident’s skill to be flexible in his/her thinking and approach depending on your needs and those of your child?	0.746**
13. Did the resident wash his/her hands?	0.367**
14. Was the resident’s identification badge visible?	0.482**
15. How would you assess the resident’s skill to pay full attention to you and your child during your interactions with him/her?	0.693**
16. How would you assess the resident’s skill to discuss what to do if your child has any problems or complications related his/her condition?	0.693**
17. How would you assess the resident’s skill to explain what he/she was doing for your child and why?	0.638**
18. How would you assess the resident’s skill to explain your child’s treatment or prescribed medication, including possible side effects?	0.400**
19. How would you assess the resident’s skill to show concern for your feelings and those of your child?	0.544**
20. How would you assess the resident’s skill to answer your questions?	0.718**
** All Pearson correlation values are significant at the 0.01 level (2-tailed).	

**Fig. 2:**
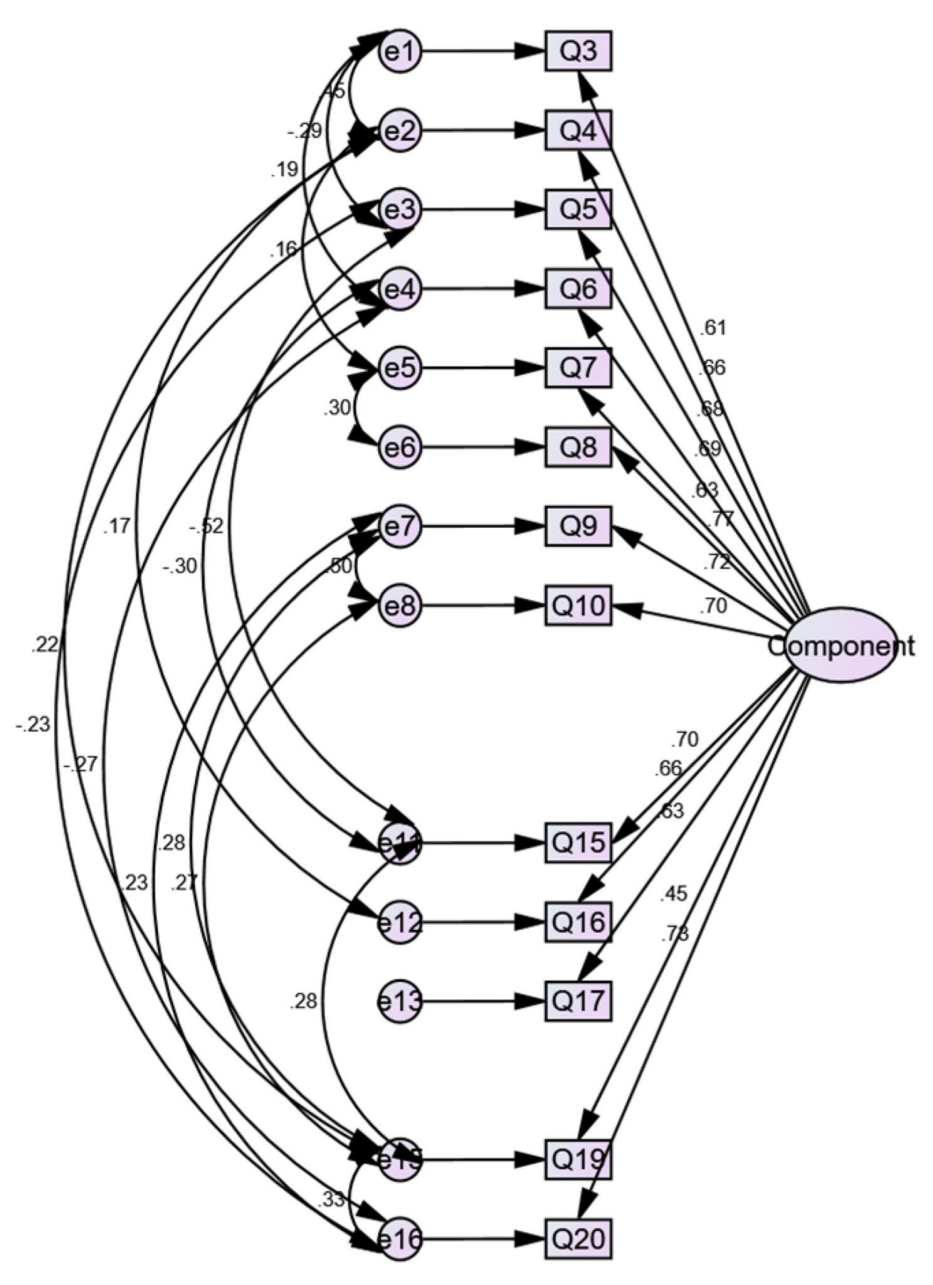
CFA Model Fit Diagram (including modification indices)

All Pearson correlation values were significant, with p values below the 0.01 threshold. This empirical finding confirmed the scale’s internal validity [19]. We further checked scale validity employing confirmatory factor analysis (CFA) using AMOS v.24 (see Fig. 2). The CFA analysis upheld a single-factor model, consistent with the exploratory factor analysis (EFA) results reported in a previous study by KA Moreau, K Eady, K Tang, M Jabbour, JR Frank, M Campbell and SJ Hamstra [11]. The goodness-of-fit indices revealed an adequate model fit: a normed chi-square (3.990) value of less than 5 shows a good fit, whereas an RMSEA (0.080) value less than 0.08 and TLI (0.932), NFI (0.945), GFI (0.941) and CFI (0.958) values greater than 0.90 indicate that the model is a good fit [20].

A composite PARENTS score was computed by averaging the scores of the 18 closed-ended items (1 = unsatisfactory to 4 = highly satisfactory). Descriptive statistics were compiled to provide an overview of the ratings (Table 4).

**Table 4:** Descriptive Statistics of PARENTS Scale Ratings

N	Minimum	Maximum	Mean	Std. Deviation	Skewness	Kurtosis
471	1.06	2.89	1.9619	0.36118	− 0.241	− 0.246

Note: Descriptive statistics for the PARENTS scale (range 1 [unsatisfactory] to 4 [highly satisfactory]). N represents the number of respondents; Standard deviation is abbreviated as Std. Deviation; Skewness and Kurtosis values are provided with their standard errors

The mean rating was 1.9619, indicating that the average performance was perceived as somewhat satisfactory. The standard deviation of 0.36118 points to a relatively tight clustering of responses around the mean. A slight negative skewness (-0.241) suggests a tendency for lower scores, while kurtosis (-0.246) indicates a fairly normal distribution of responses across the sample [21]. These statistics provide an overall assessment of PGR assessment by paediatric caregivers.

We further examined the individual PARENTS item means by grouping items into three categories based on feedback received: more than satisfactory, satisfactory, and less than satisfactory.

Caregivers rated the following five items as more than satisfactory: the resident’s ability to clearly explain concepts (item 3), their preparedness about the child’s condition before entering the room (item 4), hand hygiene adherence (item 13), visibility of the identification badge (item 14), and their capacity to concentrate entirely on the parent and child during interactions (item 15).

Six items were deemed satisfactory: identification by the resident of their role (item 2), resident’s ability to formulate follow-up plans about care or treatment (item 5), understanding of what the parents had to say (item 7), ability to interact comfortably with the parents (item 8), discussion about possible complications or problems related to the child’s condition (item 16), and explanation of their actions for the child’s benefit (item 17).

However, seven items were rated as less than satisfactory: resident’s self-introduction at first meeting (item 1), ability to listen without interrupting (item 6), comfort in interacting with the child (item 9), flexibility in thinking and approach based on the needs of the parent and child (item 10), explanation of the child’s treatment or prescribed medication including possible side effects (item 18), ability to demonstrate concern for the feelings of the parent and child (item 19), and ability to address the parents’ queries (item 20).

In response to PARENTS Item 11, an open-ended question that sought suggestions for resident improvement in their interactions with caregivers and their children, a majority of caregivers (52.9%; n = 249) offered no specific suggestions. However, some provided constructive feedback; 20.8% (n = 98) suggested that residents should communicate more politely, 14.2% (n = 67) requested residents to spend more time with them, 9.8% (n = 46) wished for the residents to display more patience, and 2.3% (n = 11) recommended residents provide more detailed information.

Regarding PARENTS Item 12, another open-ended question inviting additional comments on the resident’s skills in interaction, responses were coded into three categories. A majority of the caregivers (52.9%; n = 249) rated the residents’ skills as ‘Good’, while a considerable proportion (28.7%; n = 135) found the interactions ‘Satisfactory’. In addition, 18.5% (n = 87) of the participants expressed that the residents’ skills were ‘Excellent’.

## Discussion

In this study, we validated the Urdu translation of the PARENTS, which has proven to be a reliable and effective instrument for evaluating the NTS of PGRs in the PED of a tertiary care hospital. Through our item analysis, we identified key areas of satisfactory performance, as well as aspects that require further improvement in terms of NTS.

The Urdu version of PARENTS can be effectively used in the Pakistani environment for measuring the NTS of PGRs. This is the second use of a translated version of PARENTS in an Asian environment [12]. Consistent with earlier studies [22, 23], we found that the selection of items in PARENTS provides a useful feedback mechanism for assessing the NTS of PGRs.

Our work with the Urdu PARENTS supports the need for translated medical tools in Asian settings. This finding reinforces the relevance of previous studies in China [24] and Southeast Asia [25], demonstrating the successful adaptation and application of translated assessment tools in diverse Asian healthcare contexts.

We found that caregivers were more than satisfied with the skills of the PGRs to assess the condition of the child and to be able to explain it to the parent effectively – this also included basics such as hygiene and display of proper identification. This finding can be attributed to the basic communication skills training provided to PGRs, which has been demonstrated to positively influence patient interactions during clinical rounds [26].

Skills relating to counselling of patients were rated as satisfactory by caregivers. These items relate to skills of the PGR in being able to explain the diagnosis to the parent, the proposed treatment plan, what to expect in terms of possible side effects, and follow-ups needed. In this type of communication, the PGR must be willing to listen to the concerns of the parent. Readiness for discharge from hospital is an area of study that has in recent years started receiving attention from researchers – this is of particular importance for chronic or serious patients who need to be given confidence that their treatment can be safely continued at home [27]. A caregiver can often be worried about what to do once they leave the hospital – how to know if things are not going well, who to contact if there is a problem, etc. In this interaction, it is important that PGRs hear them out and offer the needed advice to settle their anxieties or fears.

Finally, skills in which caregivers assessed PGR as less than satisfactory were related to building rapport with caregiver/patient. These are skills that involve patience and empathy from the PGR. They are needed to gain the trust/confidence of the parent and the child. They help in getting the acceptance of the parent for the PGR to examine the child without any inhibition or resistance. By building good rapport, the PGR can help to reduce the feeling of anxiety in the parent – this is especially important in an emergency context where a patient is often brought in a serious condition [28].

Studies have shown the importance of rapport building by doctors in gaining the trust of patients to help improve treatment outcomes [29]. These sets of skills are not taught in medical education – those who have empathy by nature can develop it further during their clinical training. However, those who lack this soft skill are unlikely to develop these without the intervention of supervisors. It is in this area that we noticed a shortcoming in the NTS of the PGRs. Parent ratings were unsatisfactory – as part of the 2 open-ended questions, the respondents recommended that PGRs should show more patience when dealing with caregivers and patients. Not showing concerns for the feelings of the caregiver or child and lacking the flexibility to address their specific needs were items of particular concern. Supervisors often instruct PGRs in this area during their teaching rounds – but the feedback that we have received shows that empathy and counselling skills needed for rapport building still need much more work.

This study has provided medical educationists with a valuable tool to be incorporated in assessment strategies for soft skills. We have gained good insight into the specific areas where curriculum planners need to focus on building the soft skills of PGRs as a part of professionalism. Supervisors can use feedback from PARENTS to design and assess training approaches that can help in achieving desirable levels of NTS for PGRs to enable them to become capable clinicians.

The Urdu version of PARENTS can be used for NTS assessment as part of both undergraduate and postgraduate medical curricula during clinical rotations. This will help supervisors provide item-specific feedback to students/trainees to help them develop proper clinical attitudes and behaviours. Paediatric caregiver feedback can also be used as a useful metric as part of the annual performance report for the confirmation/promotion of PGRs, as suggested by A Dandekar, MLR Weintraub, ED McFeely and R Chasnovitz [30].

### Strengths

The study was conducted at the largest paediatric tertiary care centre (1200 beds) in Pakistan with a diverse patient population. The sample size was sufficient to evaluate the reliability and validity of the tool. Only caregivers whose patient had spent sufficient (24 h) time in the hospital were interviewed to ensure that reliable feedback on the behaviour of treating PGR could be obtained.

### Limitations

Although the collection of data from a single source (CHICH) is a limitation in this research, achieving the needed sample size for conducting statistical analysis to establish reliability and validity offsets this limitation.

## Conclusions

PARENTS has been shown to be a valid and reliable tool for measuring the NTS of PGR from the pediatric caregiver’s perspective. The items in the PARENTS provide feedback on important aspects such as resident knowledge, empathy in dealing with patients and the willingness/ability to patiently listen to and respond to patients’ concerns. Supervisors can use the Urdu version of PARENTS as an important part of their assessment tool set for their PGRs. Medical administrators can use PARENTS as a performance measurement tool for monitoring the soft side of patient care.

Further qualitative research can be conducted to identify the reasons for satisfaction or lack of satisfaction expressed by caregivers. This research can help in developing effective training strategies for medical educationists.

## Data Availability

The datasets used and/or analysed during the current study are available from the corresponding author upon reasonable request.

## Declarations

### Authors’ information (optional)

Professor Dr. Nabila Talat is Chairperson Department of Paediatric Surgery and Dean Faculty of Surgery at University of Child Health Sciences, The Children’s Hospital Lahore, Pakistan.

### Abbreviations

N/A.
